# Audience segmentation to disseminate behavioral health evidence to legislators: an empirical clustering analysis

**DOI:** 10.1186/s13012-018-0816-8

**Published:** 2018-09-19

**Authors:** Jonathan Purtle, Félice Lê-Scherban, Xi Wang, Paul T. Shattuck, Enola K. Proctor, Ross C. Brownson

**Affiliations:** 1 Department of Health Management & Policy, Dornsife School of Public Health, Drexel University, 3215 Market St., Philadelphia, PA 19104 USA; 2 Department of Epidemiology and Biostatistics, Dornsife School of Public Health, Drexel University, Philadelphia, PA USA; 3 A.J. Drexel Autism Institute, Drexel University, Philadelphia, PA USA; 4 Center for Mental Health Services Research, Brown School of Social Work, Washington University in St. Louis, St. Louis, MO USA; 5 Prevention Research Center in St. Louis, Brown School of Social Work, Washington University in St. Louis, St. Louis, MO USA; 6 Division of Public Health Sciences and Alvin J. Siteman Cancer Center, Washington University School of Medicine, Washington University in St. Louis, St. Louis, MO USA

**Keywords:** Dissemination, Audience segmentation, Policymaker, State legislators, Latent class analysis, United States

## Abstract

**Background:**

Elected officials (e.g., legislators) are an important but understudied population in dissemination research. Audience segmentation is essential in developing dissemination strategies that are tailored for legislators with different characteristics, but sophisticated audience segmentation analyses have not been conducted with this population. An empirical clustering audience segmentation study was conducted to (1) identify behavioral health (i.e., mental health and substance abuse) audience segments among US state legislators, (2) identify legislator characteristics that are predictive of segment membership, and (3) determine whether segment membership is predictive of support for state behavioral health parity laws.

**Methods:**

Latent class analysis (LCA) was used. Data were from a multi-modal (post-mail, e-mail, telephone) survey of state legislators fielded in 2017 (*N* = 475). Nine variables were included in the LCA (e.g., perceptions of behavioral health treatment effectiveness, mental illness stigma). Binary logistic regression tested associations between legislator characteristics (e.g., political party, gender, ideology) and segment membership. Multi-level logistic regression assessed the predictive validity of segment membership on support for parity laws. A name was developed for each segment that captured its most salient features.

**Results:**

Three audience segments were identified. Budget-oriented skeptics with stigma (47% of legislators) had the least faith in behavioral health treatment effectiveness, had the most mental illness stigma, and were most influenced by budget impact. This segment was predominantly male, Republican, and ideologically conservative. Action-oriented supporters (24%) were most likely to have introduced a behavioral health bill, most likely to identify behavioral health issues as policy priorities, and most influenced by research evidence. This was the most politically and ideologically diverse segment. Passive supporters (29%) had the greatest faith in treatment effectiveness and the least stigma, but were also least likely to have introduced a behavioral health bill. Segment membership was a stronger predictor of support for parity laws than almost all other legislator characteristics.

**Conclusions:**

State legislators are a heterogeneous audience when it comes to behavioral health. There is a need to develop and test behavioral health evidence dissemination strategies that are tailored for legislators in different audience segments. Empirical clustering approaches to audience segmentation are a potentially valuable tool for dissemination science.

**Electronic supplementary material:**

The online version of this article (10.1186/s13012-018-0816-8) contains supplementary material, which is available to authorized users.

## Background

When informed by research evidence, legislators’ decisions can radically accelerate the population impact of mental health and substance abuse research (hereafter referred to as behavioral health) [1–6]. For example, evidence about the benefits of insurance regulations (e.g., state parity laws) [7, 8], effective treatments, preventive interventions, and macro determinants of behavioral health (e.g., structural stigma) [9, 10] can be translated into action through laws that the 7383 state legislators in the USA have the exclusive authority to enact [11]. For these reasons, the importance of disseminating behavioral health evidence to legislators has been articulated by actors ranging from behavioral health professionals [12] to the US National Academies [13, 14]. Although the rationale for disseminating behavioral health evidence to legislators is clear, there is sparse empirical guidance about how to effectively do it [6, 15–17].

Policy dissemination research—defined as the study of the targeted distribution of evidence to policymakers [18]—has almost exclusively focused on physical health [16, 19, 20]. A 2015 review of interventions to increase the use of mental health research in policymaking found few studies, and none focused on legislators [15]. A review of policy dissemination research funded by the National Institutes of Health between 2007 and 2014 did not identify any projects focused on disseminating behavioral health evidence to legislators or other policymakers in the USA [16]. Effective policymaker-focused dissemination strategies are presumably different for behavioral health than physical health because stigma towards people with behavioral health conditions is pervasive [13, 21–26] and because willingness to allocate financial resources is lower for behavioral health than physical health services [27, 28]. Furthermore, while policy dissemination research is a rapidly growing field outside of the USA [18], these studies have primarily focused on administrative officials who have specialized knowledge about health. Elected policymakers, such as legislators, generally lack such knowledge and thus have distinct dissemination needs [6]. In the absence of empirical evidence about how behavioral health research might be most effectively disseminated to elected policymakers, dissemination activities are typically based on conjecture about what will work and an assumption about how information might be perceived by policymakers with different characteristics.

 As Leeman and colleagues note in their typology of implementation strategies, *audience segmentation research* is an essential first step towards understanding how to effectively disseminate evidence to legislators or any target population [29]. Audience segmentation theory is founded on the premise that a population’s members are heterogeneous in their knowledge and attitudes and that tailored dissemination strategies that reflect these differences are more effective and persuasive than “one-size-fits-all” dissemination [30]. Routine in the fields of marketing and communication, the practice of audience segmentation entails formative assessment to identify discrete sub-populations (i.e., audience segments) that are similar in their attitudes and behaviors [31]. Messages that are tailored to audience segments are generally more effective than non-tailored messages [32, 33]. Despite the importance of audience segmentation, policymakers have typically been treated as a homogenous population in policy dissemination research in the USA [18] and sophisticated audience segmentation analyses have not been conducted.

There are two main approaches to audience segmentation: demographic separation and empirical clustering [34]. With demographic separation, a population is divided into audiences on the basis of demographic characteristics (e.g., gender, political party affiliation) with the assumption that there is homogeneity within demographic groups. A more sophisticated approach, empirical clustering, uses statistical techniques (e.g., latent class analysis, *k*-means clustering) to identify audience segments based on relationships among relevant variables. Empirical clustering is considered the superior approach [34] and has been used to identify audience segments among the general public related to issues such as climate change [35–39] and health equity [40]. To our knowledge, no studies have used an empirical clustering approach to identify audience segments related to behavioral health among the general public or policymakers. More broadly, empirical clustering approaches to audience segmentation have not been widely used in the field of dissemination science.

## Study aims and purpose

We conducted an empirical clustering audience segmentation study using latent class analysis (LCA) of data from a survey of US state legislators. The aims were to:

1. Identify behavioral health audience segments among state legislators
2. Identify legislator characteristics that are predictive of segment membership
3. Determine whether segment membership is predictive of support for state behavioral health parity laws

The purpose of the study was to inform the design of dissemination strategies that are tailored for legislators in different audience segments, with ultimate goal of improving evidence-informed behavioral health policymaking among state legislators. It should be emphasized, however, that the use of research evidence in policymaking is extremely complex and that there are myriad barriers to evidence-informed policymaking (e.g., lack of trust in researchers, lack of policy relevant research findings, limited capacity to use research) [19, 41–44]. Audience segmentation, or improved dissemination more broadly, is not a solution to these issues [45, 46]. Rather, it simply provides an empirical basis for packaging and communicating research evidence in ways that more accurately reflect policymakers’ characteristics.

## Methods

### Study design and data

Between March and September 2017, we conducted a multi-modal (post-mail, e-mail, telephone) survey of US state legislators (study protocol published in *Implementation Science*) [47]. To construct the sample frame, we used the National Conference of State Legislatures’ (NCSL) database and randomly selected up to 60 legislators from every state who were legislators as of January 15, 2017. We excluded legislators who had just entered office on January 1, 2017, because they would have limited legislative experience at the time of survey completion. After excluding legislators whose contact information was invalid and those who left office prior to when data collection began, the sample frame consisted of 2902 legislators.

Each legislator received a post-mailed invitation to complete the survey online, two post-mailed paper versions of the survey with self-addressed postage-paid return envelopes, ten e-mail invitations to complete the survey online, and up to 15 telephone calls. In total, a legislator who did not complete the survey was contacted 29 times. Recruitment materials stated that only the legislator, not their staff, could complete the survey. Data were collected by a survey research firm, and Institutional Review Board approval was obtained.

The survey was completed by 475 legislators, and the response rate was 16.4%, which is reasonable for state legislators [48] and is higher than response rates of other recent state legislator surveys [49–51]. Respondents were significantly more likely than non-respondents to be Democrat (48.8% vs. 42.4%, *χ*^2^ = 10.19, *p* = .001), female (32.6% vs. 23.0%, *χ*^2^ = 19.73, *p* < .001), and from the Midwest US Census Region (30.5% vs. 22.5%, *χ*^2^ = 14.27, *p* < .001). To account for these differences and make our results more generalizable to the entire population of state legislators, we calculated and applied non-response weights accounting for political party, gender, and geographic region using a sample post-stratification approach in which weighting classes were based on the full sampling frame [52]. Weighted and unweighted results were only modestly different, suggesting that results were not strongly driven by legislator characteristics specific to the sample.

### Variables and analysis

The survey instrument (Additional file 1) contained 59 items and was vetted by NCSL and cognitively pre-tested with five former state legislators prior to fielding. Most survey items were adapted from questions previously used in public opinion surveys about behavioral health issues or legislator surveys about research use in policymaking [47].

Consistent with recommendations for cognitive pre-testing [53], the draft survey was e-mailed to former state legislators and telephone-based interviews were conducted to assess whether questions and response options were clear. The main finding from these interviews was that mental health and substance use disorders were through of separate, although often related, issues. Thus, the survey was revised to explicitly anchor questions to “mental health” and/or “substance use disorder” issues as opposed to only using language of “mental health” or “behavioral health.”

#### LCA variables

Nine variables that spanned five domains were included in the LCA. All variables were dichotomous to aide interpretability. Variable selection was informed by Watson and Corrigan’s framework of factors that influence policymakers’ behavioral health policy decisions [5] and McGinty and colleagues’ review of communication strategies to reduce stigma and generate support for behavioral health policies [22]. We did not seek to map variables onto the framework or review, but rather used these sources to orient the selection of variables that are likely to be associated with legislators’ behavioral health policymaking behaviors.

##### Perceptions of behavioral health treatment effectiveness (two variables)

Legislators separately rated the extent to which they agreed that “mental health treatments can help people with mental illness lead normal lives” and “substance disorder treatments can help people with a substance use disorder recover” (1 = “strongly disagree,” 5 = “strongly agree”). The mental health treatment effectiveness question has been previously used in Behavioral Risk Factor Surveillance System surveys [54] and was adapted by the authors to assess perceptions of substance use disorder treatment effectives. These variables were dichotomized as “strongly agree,” yes/no.

##### Stigma towards people with mental illness (one variable)

Stigma was assessed using a composite measure based on four items used by Barry and McGinty [23] to assess stigma among the US general public. Two items assessed attitudes about the dangerousness of people with mental illness [55] and two assessed preferences for social distance from people with mental illness [56]. Cronbach’s alpha for the four items was .80, similar to when the four items were used with the general public (Cronbach’s alpha = .77) [23]. We coded responses so that higher scores corresponded with greater stigma and summed them to create a stigma score for each respondent (range = 0 to 16). In the absence of a priori theoretically or empirically relevant cut-points, we classified legislators based on sample quartiles of the score.

##### Factors that have the most influence on support for a behavioral health bill (two variables)

Legislators selected up to two factors, from a list of five options, that “have the most influence on whether [they] support” a “mental health/substance abuse bill” when it is introduced. The list of factors was developed by the authors, informed by literature about decision making among state legislators [57]. We classified each legislator according to whether or not they selected “the extent to which the bill is going to impact the state budget” and “is based on scientific evidence.”

##### Most important health issues for legislative action in the state (two variables)

Legislators were presented with a list of 19 health issues and selected up to three that were most important “for legislative action in [their] state.” This question and the list of health issues have been previously used in surveys of state legislators [58, 59]. We classified each legislator according to whether they selected “mental health” or “substance abuse,” respectively. We also reviewed open-ended response options and coded responses accordingly (e.g., coded “access the mental health services” as mental health and coded “opioid epidemic” as substance abuse).

##### History of introducing a behavioral health bill (two variables)

Legislators separately indicated whether they had ever introduced a “mental health” or “substance abuse” bill.

#### Identification of audience segments

Latent class analysis (LCA) is considered the optimal analytic strategy for audience segmentation [60]. We used the SAS PROC LCA add-on [61] to identify audience segments of legislators based on the clustering of their responses to the nine variables that were selected for inclusion in the LCA [62]. We fit models using three through five segment solutions and then selected the most appropriate model based on the criteria of interpretability, identification, and fit statistics. Fit statistics included the adjusted Aikake Information Criterion, Bayesian Information Criterion (BIC), and sample-size-adjusted BIC [63]. To assess model identification, we estimated the parameters for each model using 1000 random starting values and measured the proportion of iterations that converged to the same maximum likelihood solution [62]. Based on these criteria, we found that a three segment solution was optimal (Table 1). We then developed a name for each segment that concisely captured its most salient and distinct features [37].

**Table 1:** Latent class analysis fit statistics

Number of segments	Log-likelihood	AIC	BIC	Adjusted BIC	Degree of freedom	Identification (1000 starting values)
3	− 2924.46	895.61	1041.33	930.24	988	100%
4	− 2896.03	862.74	1058.42	909.25	976	60.2%
5	− 2874.15	843.00	1088.63	901.38	964	33.0%

*AIC* Aikake Information Criterion, *BIC* Bayesian Information Criterion

#### Identification of predictors of segment membership

In order to test predictors of segment membership, we assigned each legislator to the segment for which they had the highest posterior probability of membership. We then used binary logistic regression to test associations of legislator characteristics with membership in the three segments. We modeled membership in each class segment as a separate model, using the other two segments combined as the referent group.

For each segment, we used a sequential modeling approach in which we first included legislator characteristics for which information is readily and publicly available (i.e., political party, gender, current health committee membership, number of years serving as a legislator, and US Census Region). Then, in a second model, we added characteristics that would typically require additional primary data collection (i.e., social and fiscal ideology, highest level of education). Social and fiscal ideologies were assessed separately in the survey using items adapted from American National Election Studies’ questionnaires [64] that have been previously used with state legislators [65].

We used odds ratios and 95% confidence intervals (CIs) to quantify associations of each potential predictor with class membership after adjustment for the other variables in the model. Because our method of assigning participants to discrete segments did not account for uncertainty in segment membership, we conducted a sensitivity analysis in which the legislator characteristic variables were included as covariates in the estimation of the LCA [62]. Results were consistent, and we present the results using separate logistic regression models for ease of interpretability.

#### Determining the predictive validity of segment membership

To assess the predictive validity of segment membership on behavioral health policy support, we used multi-level logistic regression to examine associations between segment membership and support for state behavioral health parity laws. The multi-level models used state-level random intercepts to account for correlated responses of legislators from the same state. We focused on parity laws because they are an evidence-based policy recommended by the US Task Force on Community Preventive Services [7, 8]. A definition of behavioral health parity laws was provided (see Additional file 1), and legislators rated the extent to which they supported them (1 = “strongly oppose,” 5 = “strongly support”). This variable was dichotomized as “strongly support,” yes/no. We ran unadjusted models as well as models adjusted for legislator characteristics. The adjusted models allowed us to determine whether segment membership was predictive of policy support independent of these covariates and compare predictive power of segment membership relative to non-latent variables.

## Results

### Characteristics of audience segments and predictors of segment membership

Table 2 presents data on the distribution of the nine LCA variables across the three segments, Table 3 shows the demographic characteristics of legislators in each segment, and Table 4 presents logistic regression results showing adjusted associations between each demographic characteristic and segment membership. The narrative below describes the characteristics of each of the audience segments.

**Table 2:** Distribution of the 12 latent class analysis variables across the three audience segments, US state legislators, 2017 (*N* = 475)

	All	Budget-oriented skeptics with stigma	Action-oriented supporters	Passive supporters	*χ*^2^ *p* value
	%	%	%	%	
Perceptions of behavioral health treatment effectiveness					
Strong agreement that mental health treatments can be effective	54.1	16.9	73.8	98.9	< .0001
Strong agreement that substance use disorder treatments can be effective	49.1	12.6	78.5	84.8	< .0001
Mental illness stigma score quartile					
1st quartile (score range = 0, 3)	30.5	12.0	47.1	46.6	< .0001
2nd quartile (score range = 4, 5)	17.8	11.2	19.6	27.1	
3rd quartile (score range = 6, 8)	30.9	42.6	23.2	18.5	
4th quartile (score range = 9, 14)	20.7	34.2	10.1	7.8	
Factors that have the most influence on support for a behavioral health bill					
Extent to which the bill is going to impact the state budget	47.7	61.4	29.2	40.5	< .0001
Extent to which the bill is based on scientific evidence	60.5	46.1	74.1	72.7	< .0001
Most important health issues for legislative action in the state					
Mental health	37.1	29.3	45.6	43.0	.0007
Substance abuse	45.0	41.1	58.3	40.2	.004
History of introducing behavioral health bill					
Mental health bill	34.8	13.4	90.7	23.2	< .0001
Substance abuse bill	31.4	15.4	96.3	4.6	< .0001

*χ*^2^ testing differences in the proportion of legislators with each latent class analysis variable characteristic across audience segments

**Table 3:** Demographic characteristics of the three audience segments, US state legislators, 2017 (*N* = 475)

	All	Budget-oriented skeptics with stigma	Action-oriented supporters	Passive supporters	*χ*^2^ *p* value
	%	%	%	%	
Gender					
Female	24.6	16.2	29.7	34.2	.0002
Male	75.4	83.8	70.3	65.9	
Political party					
Democrat	43.5	24.9	51.5	66.9	< .0001
Other	2.5	1.5	3.1	3.4	
Republican	54.1	73.6	45.4	29.7	
Geographic region					
West	24.0	23.5	23.6	25.2	.002
Midwest	19.0	12.7	21.6	26.9	
South	32.0	39.4	32.3	19.8	
Northeast	25.0	24.4	22.4	28.1	
Current health committee member					
No	61.9	70.8	39.2	66.0	< .0001
Yes	38.1	29.2	60.8	34.0	
Years as legislator					
≤ 5	46.7	51.6	29.1	53.1	< .0001
≥ 6	53.3	48.4	70.9	46.9	
Social ideology					
Conservative	45.3	66.1	34.8	20.3	< .0001
Moderate	35.2	17.0	41.9	59.3	
Liberal	19.5	17.0	23.3	20.4	
Fiscal ideology					
Conservative	59.6	78.3	51.8	35.9	< .0001
Moderate	21.8	9.2	28.8	36.5	
Liberal	18.6	12.5	19.4	27.6	
Education					
≤ College	51.3	56.8	40.1	51.7	.016
≥ Postgraduate	48.7	43.2	59.9	48.3	

*χ*^2^ testing differences in demographic characteristics across audience segments

**Table 4:** Adjusted associations between demographic characteristics and audience segment membership, binary logistic regression, US state legislators, 2017 (*N* = 475)

	Budget-oriented skeptics with stigma		Action-oriented supporters		Passive supporters	
	Model 1	Model 2	Model 1	Model 2	Model 1	Model 2
	aOR	aOR	aOR	aOR	aOR	aOR
Gender						
Female	Ref.	Ref.	Ref.	Ref.	Ref.	Ref.
Male	1.79 (1.09, 2.94)*	1.72 (1.03, 2.87)*	0.80 (0.48, 1.35)	0.82 (0.48, 1.39)	0.71 (0.44, 1.15)	0.73 (0.45, 1.20)
Political party						
Republican	Ref.	Ref.	Ref.	Ref.	Ref.	Ref.
Democrat	0.21 (0.14, 0.33)*	0.61 (0.30, 1.25)	1.53 (0.94, 2.48)	1.27 (0.57, 2.84)	4.17 (2.62, 6.65)*	1.56 (0.72, 3.34)
Other	0.21 (0.05, 0.89)*	0.56 (0.12, 2.68)	2.35 (0.55, 10.10)	1.78 (0.37, 8.52)	2.69 (0.74, 9.78)	1.17 (0.28, 4.92)
Region						
West	Ref.	Ref.	Ref.	Ref.	Ref.	Ref.
Midwest	0.71 (0.39, 1.29)	0.65 (0.35, 1.21)	1.46 (0.73, 2.90)	1.66 (0.82, 3.37)	1.11 (0.60, 2.05)	1.08 (0.57, 2.06)
Northeast	0.56 (0.30, 1.07)	0.62 (0.32, 1.21)	1.50 (0.75, 3.01)	1.44 (0.70, 2.96)	1.18 (0.64, 2.16)	1.06 (0.57, 1.98)
South	1.67 (0.97, 2.89)	1.59 (0.90, 2.81)	1.06 (0.57, 1.97)	1.00 (0.53, 1.89)	0.52 (0.29, 0.94)*	0.58 (0.31, 1.08)
Current health committee member						
No	Ref.	Ref.	Ref.	Ref.	Ref.	Ref.
Yes	0.34 (0.22, 0.53)*	0.34 (0.21, 0.53)*	3.80 (2.38, 6.06)*	3.94 (2.44, 6.37)	0.91 (0.58, 1.43)	0.87 (0.55, 1.38)
Years as legislator						
≤ 5	Ref.	Ref.	Ref.	Ref.	Ref.	Ref.
≥ 6	0.68 (0.45, 1.03)	0.66 (0.43, 1.02)	2.80 (1.73, 4.54)*	2.78 (1.70, 4.53)*	0.63 (0.41, 0.98)*	0.63 (0.40, 0.99)*
Social ideology						
Conservative	–	Ref.	–	Ref.	–	Ref.
Moderate	–	0.43 (0.23, 0.79)*	–	1.64 (0.82, 3.27)	–	2.23 (1.12, 4.46)*
Liberal	–	0.40 (0.18, 0.89)*	–	1.07 (0.44, 2.58)	–	2.94 (1.25, 6.90)*
Fiscal ideology						
Conservative	–	Ref.	–	Ref.	–	Ref.
Moderate	–	0.68 (0.35, 1.35)	–	0.91 (0.42, 1.96)	–	1.53 (0.77, 3.06)
Liberal	–	0.44 (0.20, 0.98)*	–	1.46 (0.63, 3.37)	–	1.41 (0.67, 2.98)
Education						
≥ Postgraduate	–	Ref.	–	Ref.	–	Ref.
≤ College	–	1.53 (0.99, 2.38)	–	0.55 (0.34, 0.90)*	–	1.07 (0.68, 1.68)

*aOR* adjusted odds ratio, *CI* confidence interval

*p ≤ .05 Model 1 adjusted for gender, political party, region, current health committee membership, and years as legislator. Model 1 adjusted for gender, political party, region, current health committee membership, and years as legislator. Model 2 adjusted for gender, political party, region, current health committee membership, years as legislator, social ideology, fiscal ideology, and education

#### Budget-oriented skeptics with stigma

This constituted the largest segment of legislators (47%). Legislators in this segment were the most skeptical about the potential effectiveness of mental health and substance abuse treatments—with only 16.9% and 12.6%, respectively, strongly agreeing that treatments can help people lead normal lives and recover (Table 2). Legislators in this segment also had much more stigma towards people with mental illness than legislators in the other segments, with 34.2% having stigma scores in the fourth quartile (most stigma) and 42.6% in the third quartile. Among legislators in this segment, more reported that the impact that a behavioral health bill will have on the state budget (61.4%) influenced their support than the extent to which the bill is based on evidence (46.1%).

Unlike legislators in the other two segments, the majority of legislators in this segment were Republican (73.6%), socially conservative (66.1%), and fiscally conservative (78.3%) (Table 3). However, political party was not significantly associated with segment membership after adjustment for all other predictors (Table 4, model 2). This was also the most predominantly male segment (83.8%). After adjustment, the odds of a male legislator being in this segment was nearly twice that of a female (Table 4; aOR = 1.72, 95% CI = 1.03, 2.87).

#### Action-oriented supporters

This was the smallest segment of legislators (24%). Legislators in this segment were, by far, the most likely to have introduced a behavioral health bill, with 90.7% having introduced a mental health bill and 96.3% having introduced a substance abuse bill (Table 2). Legislators in this segment were also the most likely to identify substance abuse issues as policy priorities (58.3%) and nearly half (45.6%) also prioritized mental health issues. These legislators were more likely to report that the extent to which a bill was based on evidence (74.1%) influenced whether they supported a behavioral health bill than the impact it would have on the state budget (29.2%).

Unlike legislators in the other two segments, the majority of legislators in this segment were on a health committee (60.8%) (Table 3). After adjustment, health committee members had nearly four times the odds of being in this segment as legislators who were not health committee members (aOR = 3.94, 95% CI = 2.44, 6.37) (Table 4, model 2). Legislators in this segment also were more likely to have been long-time legislators, with 70.9% having been a legislator for ≥ 6 years compare to less than half of the legislators in the other two segments (Table 3). After adjustment, the odds of a legislator with ≥ 6 years of experience being in this segment was nearly three times as that of a legislator with < 6 years of experience (aOR = 2.78, 95% CI = 1.70, 4.53) (Table 4, model 2). This was also the most ideologically diverse segment, with the modal political party being democrat (51.5%), the modal social ideology being moderate (41.9%), and the modal fiscal ideology being conservative (51.8%) (Table 3).

#### Passive supporters

Twenty-nine percent of legislators belonged to this segment. Legislators in this segment had the most faith in treatment effectiveness with 98.9% and 84.8%, respectively, strongly agreeing that treatments can help people lead normal lives and recovery (Table 2). These legislators also had the least stigma towards people with mental illness with only 7.8% having stigma scores in the fourth quartile (most stigma) and 18.5% in the third quartile. Despite their faith in the effectiveness of behavioral health treatments and low levels of stigma, a relatively small proportion had introduced a mental health bill (23.2%) and barely any (4.6%) had introduced a substance abuse bill.

This was the segment with the most gender diversity (34.2% female, 65.9% male; Table 3). It was also the segment with the largest proportion of Democrats (66.9%), and over one fifth identified as fiscally and socially liberal (27.6% and 20.4%, respectively). After adjustment, the odds of a socially liberal legislator being in this segment were nearly three times that of a socially conservative legislator (aOR = 2.94, 95% CI = 1.25, 6.90) (Table 4, model 2).

### Predictive validity of segment membership on support for state parity laws

Table 5 shows unadjusted and adjusted associations of segment membership with strong support for behavioral health parity laws. The proportion of legislators strongly supporting state behavioral health parity laws was lowest in the budget-oriented skeptics with stigma segment (15.8%) and similar between the action-oriented proponent (66.4%) and passive believer segments (65.1%). After adjustment for legislator characteristics and state-level correlations, the odds of strong support for state parity laws were three times higher among passive supporters (aOR = 3.47, 95% CI = 1.83, 6.60) and more than six times higher among action-oriented supporters (aOR = 6.67, 95% CI = 3.30, 13.46) than budget-oriented skeptics with stigma (Table 5). Importantly, segment membership was a stronger predictor of strong support for state parity laws than almost all other socio-demographic characteristics.

**Table 5:** Adjusted associations between audience segment membership, demographic characteristics, and strong support for state behavioral health parity laws. Multi-level binary logistic regression, US state legislators, 2017 (*N* = 475)

	AOR (95% CI)
Audience segment	
Budget-oriented skeptics with stigma	Ref.
Passive supporters	3.47 (1.83, 6.60)*
Action-oriented supporters	6.67 (3.30, 13.46)*
Gender	
Female	Ref.
Male	0.87 (0.48, 1.57)
Political party	
Republican	Ref.
Democrat	3.30 (1.41, 7.71)*
Other	2.91 (0.46, 18.25)
Region	
West	Ref.
Midwest	1.61 (0.64, 4.08)
Northeast	1.48 (0.58, 3.78)
South	0.94 (0.40, 2.21)
Current health committee member	
No	Ref.
Yes	1.79 (1.01, 3.18)*
Years as legislator	
≤ 5	Ref.
≥ 6	0.78 (0.45, 1.34)
Social ideology	
Conservative	Ref.
Moderate	2.90 (1.35, 6.26)*
Liberal	7.22 (2.74, 18.98)*
Fiscal ideology	
Conservative	Ref.
Moderate	0.87 (0.39, 1.91)
Liberal	0.96 (0.40, 2.33)
Education	
≥ Postgraduate	Ref.
≤ College	1.25 (0.73, 2.15)

*aOR* adjusted odds ratio, *CI* confidence interval

*p ≤ .05. Adjusted for gender, political party, region, current health committee membership, years as legislator, social ideology, fiscal ideology, and education. Multi-level regressions (state as higher level and legislators as lower level) with state-level random intercepts which accounted for correlated responses of legislators from the same state

## Discussion

Our results demonstrate that US state legislators are heterogeneous in their knowledge and attitudes about behavioral health and that at least three distinct audience segments exist: *budget-oriented skeptics with stigma* who have the least faith in behavioral health treatment effectiveness, have the most mental illness stigma, are most influenced by budget impact, and are ideologically conservative; *action-oriented supporters* who are most likely to have introduced a behavioral health bill, are most likely to identify behavioral health issues as policy priorities, are most influenced by research evidence, and are ideologically diverse; and *passive supporters* who have the greatest faith in behavioral health treatment effectiveness and the least mental illness stigma, but are also least likely to have introduced a behavioral health bill.

Membership in these latent audience segments had much higher predictive validity for support of state behavioral health parity laws than non-latent legislator characteristics (e.g., political party, education level). These findings underscore the importance of, and provide an empirical foundation for, developing and testing behavioral health dissemination strategies that are tailored to these different audience segments of legislators.

### Implications for tailored dissemination strategies

Budget-oriented skeptics with stigma are a priority population to target given that they are the largest segment of legislators (47%) and have the highest levels of mental illness stigma and the least faith in behavioral health treatment effectiveness. As these are likely the political actors who contribute to structural stigma through policies that restrict opportunities for people with mental illness [9, 10], there is an urgent need to disseminate information that reduces stigma among these legislators. Effective communications interventions to reduce stigma among the general public exist [13, 22, 66], and research is needed to understand how these interventions might be tailored for legislators in this segment. These dissemination efforts should be tailored to the conservative characteristics of this segment (e.g., 73.6% Republican, 66.1% socially conservative). Dissemination might be most effective if information is framed in ways that resonate with a conservative worldview [67, 68] and if messages originate from sources that conservative legislators perceive as credible. Post hoc analyses (Additional file 2) revealed that legislative staff and state behavioral health agencies were the primary sources that legislators in this segment turned to for behavioral health research when making policy decisions. Thus, these sources might be important intermediaries to target in dissemination efforts.

Less than 20% of legislators in the budget-oriented skeptics with stigma strongly agreed that behavioral health treatments were effective, compared to over 70% of legislators in the other two segments. As Watson and Corrigan note, legislators’ perceptions of behavioral health treatment effectiveness often represent concerns about wasting finite resources [5]. This is consistent with our findings that legislators in this segment were much more influenced by budget impact when making behavioral health policy decisions than any other group and were by far the most fiscally conservative (78.3%). Thus, messages targeting this segment might emphasize the costs of unaddressed behavioral health problems and the potential return on investment for prevention and treatment [14, 69–71]. However, it should be noted that socially conservative ideology, in addition to fiscally conservative ideology, was a strong predictor of segment membership in the fully adjusted model. This suggests that fiscal concerns are not the only core attribute of this segment. Attitudes towards people with mental illness (e.g., perceptions of the extent to which their problems are the result of individual versus structural issues) [5, 26, 72] and other characteristics often associated with people with mental illness (e.g., low social class, minority race/ethnicity) [73] potentially play an important role and should be considered in dissemination strategies.

Although action-oriented supporters was the smallest segment of legislators (24%), it is promising that this segment is characterized by prioritizing behavioral health issues, introducing behavioral health bills, and being strongly influenced by evidence when making behavioral policy decisions. It is also encouraging that this was the most politically and ideological diverse segment (e.g., 51.5% Democrat, 45.4% Republican). These findings are consistent with a 2012 survey of state legislators that found bipartisan support for behavioral health issues and that legislators who prioritized behavioral health issues were more influenced by research evidence than legislators’ who did not prioritize these issues [58]. Taken together, these findings suggest that dissemination efforts targeting legislators in the action-oriented supporters segment should include concrete information about the science supporting evidence-based policy options to address behavioral health issues. Post hoc analyses (Additional file 2) showed that legislators in this segment identified behavioral health advocacy organizations as their primary source of behavioral health research. Thus, these organizations should be a target for the dissemination of this information. Passive supporters might be the lowest priority population to target given their exceptionally high faith in behavioral health treatment effectiveness and relatively low levels of mental illness stigma. Although legislators in this segment are least likely to have introduced behavioral health bills, they are similarly influenced by research evidence as action-oriented supporters when deciding whether to support behavioral health bill. Research should assess whether legislators in this segment respond similarly to dissemination strategies that are tailored for legislators in the action-oriented supporters segment.

### Implications for knowledge translation

The current study focused on generating information to enhance the precision of dissemination efforts that *push* research evidence to legislators. Our results, however, also have potential implications for knowledge translation efforts more broadly [74]. Specifically, there could be implications for efforts that facilitate the *pull* of research by legislators in the budget-oriented skeptics with stigma segment and *exchange* efforts that foster relationships between behavioral health researchers and legislators in this segment.

In terms of *pull* efforts, evidence clearinghouses could include economic evaluation data given that considerations related to budget impact were of high importance to legislators in this segment. The Washington State Institute for Public Policy’s Benefit-Costs Results clearinghouse is one model that could be adapted in states with conservative legislatures [75]. In terms of implications for *exchange* efforts, interventions that foster positive relationships between behavioral health researchers and legislators in the budget-oriented skeptics with stigma category could increase trust in researchers and potentially increase research use. Post hoc analyses (Additional file 2) showed that only 18.3% of legislators in this segment identified universities as a primary source of behavioral health research, a proportion significantly lower than the other two segments. This is consistent with the results of a 2012 state legislator survey which found that social and fiscal conservative ideology—strong predictors of membership in the budget-oriented skeptics with stigma—was inversely associated with the extent to which universities were perceived as reliable sources of research [76]. Trusted intermediary organizations such as the American Legislative Exchange Council—an ideologically conservative legislator assistance organization—could help broker these relationships. A knowledge exchange intervention that recently demonstrated success with federal legislators in the USA is a model that could be adapted [77].

### Implications for dissemination science and future research

Our study demonstrates the utility of empirical clustering approaches to audience segmentation in policy dissemination research. Compared to prior studies that used demographic separation approaches to identify audience segments of legislators [58, 59, 65, 78], our empirical clustering approach produced a more nuanced understanding of how evidence about a specific issue (i.e., behavioral health) might be most effectively packaged for different types of legislators. Although our study was focused on legislators in the USA, dissemination studies targeting administrative (i.e., not elected) policymakers, such as those being conducted in Australia [79] and Canada [80], might consider how empirical clustering could be used to identify audience segments within government agencies.

The value of identifying audience segments hinges upon the extent to which dissemination strategies that are tailored for these segments are more effective than non-tailored strategies. Narratives (i.e., stories about people) are a medium that can be integrated into dissemination materials, tailored for different audience segments, and manipulated by researchers to test dissemination effects. Narratives are important in policymaking processes because they are engaging and evocative, can humanize abstract problems, and illustrate how contextual factors (that can often be modified by policies) affect individuals [81, 82]. Two experiments have tested the effects of narrative-focused dissemination materials on support for evidence-based policies among state legislators (one study focused on cancer [65], one focused on obesity [50]), and numerous studies have tested the effects of narratives about behavioral health issues on policy support among the general public [26, 83–85].

For example, a recent public opinion experiment by McGinty et al. [26] tested the effects of narratives that framed issues related to people with mental illness in different ways. The study found that narratives that emphasized systematic barriers to mental health treatment were more effective at increasing public willingness to pay additional taxes to improve the mental health system than a narrative about successful treatment-and-recovery. As McGinty et al. note (p. 212), future research should build on these studies and test the effects of such narratives on policymakers as opposed to the general public. By identifying behavioral health audience segments of state legislators, the current study could inform the tailoring and enhance the precision of policymaker-targeted narratives about behavioral health issues.

Measuring the effects of dissemination strategies on policymakers, particularly legislators, can be challenging [18, 86]. Proximal measures of the effectiveness of tailored versus non-tailored dissemination materials could include perceptions of the materials (e.g., perceived likelihood of using the information, clarity, and relevance) and support for evidence-based policies that are the focus of the dissemination materials. Such outcomes have been previously assessed among state legislators via brief surveys that accompany materials [50, 87, 88].

More distal measures of effectiveness could include legislators’ research use and policymaking behaviors. Information on these outcomes could be obtained via unobtrusive measures such as legislative voting [89–91], committee hearings [92, 93], the content of bills and other legislative documents [94], public statements [95], and social media behaviors [96]. Structured interviews that assess the use of research evidence in policymaking, such as the Staff Assessment of enGagement with Evidence instrument [97, 98], could also be adapted for state legislators to examine differences between those who receive tailored versus non-tailored dissemination materials.

### Limitations

Our study has five main limitations. First, while our response rate of 16.4% is reasonable for state legislators [48] and higher than response rates of recent legislator surveys [49–51], it is sub-optimal by typical health services research standards. Demographic information about non-respondents allowed us to determinate that respondents were significantly different than non-respondents in terms of their political party affiliation, gender, and geographic region and to develop and apply non-response weights to adjust for these differences. This increases our confidence that results are not biased by non-response issues.

Second, our stigma measures were focused on mental illness and not able to assess stigma towards people with substance use disorders. This distinction could be important because evidence suggests that the public holds has more negative and stigmatizing attitudes towards people with substance use disorders than mental illness [22, 24].

Third, our survey questions were broadly focused on behavioral health issues and we did not explicitly anchor questions to adult or child populations. While some of our questions implied that we were asking about adults (e.g., willingness to work closely with someone who has serious mental illness), it is not clear whether respondents were thinking of adults or children, or both, when answering questions. Perceptions of adults’ behavioral health issues, and policy solutions to address them, are often different than perceptions of children’s mental health issues [22, 99–102], and different audience segments might exist for children’s behavioral health.

Fourth, our study was limited to elected policymakers in the legislative branch of government at the state-level in the USA and results are not necessarily generalizable to elected policymakers at different levels of government, those outside the USA, or administrative policymakers in executive branches of government.

Fifth, a limitation of LCA is the risk of misclassifying of individuals, particularly those whose posterior segment membership probabilities are far from 0 or 1. In our analysis, mean posterior probabilities for segment membership ranged from 0.91 to 0.94 and mean probabilities for segment non-membership ranged from 0.03 to 0.06, which is encouraging [103].

## Conclusions

State legislators are a heterogeneous audience when it comes to behavioral health. There is a need to develop and test behavioral health evidence dissemination strategies that are tailored for legislators in different audience segments. Empirical clustering approaches to audience segmentation are a potentially valuable tool for dissemination science.

## Additional files

Additional file 1: Legislator survey instrument. (DOCX 49 kb)

Additional file 2: Source of behavioral health research across the three audience segments, US state legislators, 2017 (*N* = 475). (DOCX 48 kb)
