# Supplementary material for: Audience segmentation to disseminate behavioral health evidence to legislators: an empirical clustering analysis
Source: Implement Sci. 2018 Sep 19;13:121. doi: 10.1186/s13012-018-0816-8 (PMC6148796; doi:10.1186/s13012-018-0816-8)
Supplement: Supplementary file 1 — Legislator survey instrument. (DOCX 49 kb) [file 13012_2018_816_MOESM1_ESM.docx]

**Additional File 1: legislator Survey INSTRUMENT**

*First are five questions about comprehensive state mental health/substance use disorder parity laws. These laws are defined as state laws that require health insurance companies to provide the same level of coverage for all mental health/substance use disorder and physical health benefits (e.g., identical deductibles, copayments, visit limits) with no discrepancy.*

Qx**.** Have you heard of comprehensive state mental health/substance use disorder parity laws (defined above)?

- Yes
- No
- Not sure

Qx**.** Based the definition of comprehensive state mental health/substance use disorder parity laws provided above, to what extent do you support or oppose them?

- Strongly oppose
- Somewhat oppose
- Neither oppose nor support
- Somewhat support
- Strongly support

Qx**.** To what extent do you agree or disagree with the statement that comprehensive state mental health/substance use disorder parity laws increase access to mental health/substance use disorder services?

- Strongly disagree
- Somewhat disagree
- Neither disagree nor agree
- Somewhat agree
- Strongly agree

Qx. To what extent do you agree or disagree with the statement that comprehensive state mental health/substance use disorder parity laws increase use of mental health/substance use disorder services?

- Strongly disagree
- Somewhat disagree
- Neither disagree nor agree
- Somewhat agree
- Strongly agree

QX. To what extent do you agree or disagree with the statement that comprehensive state mental health/substance use disorder parity laws increase health insurance premium costs?

- Strongly disagree
- Somewhat disagree
- Neither disagree nor agree
- Somewhat agree
- Strongly agree

QX. Below is a list of five common features of health insurance plans. For each, indicate the extent to which you support or oppose requiring health insurance companies to provide mental health/substance use disorder coverage that is equal to physical health coverage.

- QXa. Cost of prescriptions, defined as the fixed amount a person pays for a covered prescription.
  - Strongly oppose
  - Somewhat oppose
  - Neither oppose nor support
  - Somewhat support
  - Strongly support
- QXd. Deductibles, defined as the amount a person pays for covered health care services before their insurance plan starts to pay.
  - Strongly oppose
  - Somewhat oppose
  - Neither oppose nor support
  - Somewhat support
  - Strongly support
- QXb. Co-payments, defined as the fixed amount a person pays for a covered health care service after they’ve paid their deductible.
  - Strongly oppose
  - Somewhat oppose
  - Neither oppose nor support
  - Somewhat support
  - Strongly support
- QXf. Out-of-pocket maximums, defined as the most a person pays for covered services in a year. After a person spends this amount on deductibles and copayments, their insurance plan pays 100% of the costs of covered benefits.
  - Strongly oppose
  - Somewhat oppose
  - Neither oppose nor support
  - Somewhat support
  - Strongly support
- QXh. Limits to hospital stays or outpatient treatment sessions, defined as the maximum number of days in the hospital or outpatient treatment sessions that an insurance plan will pay for.
  - Strongly oppose
  - Somewhat oppose
  - Neither oppose nor support
  - Somewhat support
  - Strongly support

QX. Below is a list of six common mental illnesses/substance use disorders. For each, indicate the extent to which you support or oppose requiring health insurance companies to provide coverage that is equal to physical coverage.

- QXa. Major depression disorder
  - Strongly oppose
  - Somewhat oppose
  - Neither support nor oppose
  - Somewhat support
  - Strongly support
- QXg. Alcohol use disorder
  - Strongly oppose
  - Somewhat oppose
  - Neither support nor oppose
  - Somewhat support
  - Strongly support
- QXc. Post-traumatic stress disorder
  - Strongly oppose
  - Somewhat oppose
  - Neither support nor oppose
  - Somewhat support
  - Strongly support
- QXh. Opioid use disorder
  - Strongly oppose
  - Somewhat oppose
  - Neither support nor oppose
  - Somewhat support
  - Strongly support
- QXe. Schizophrenia
  - Strongly oppose
  - Somewhat oppose
  - Neither support nor oppose
  - Somewhat support
  - Strongly support
- QXf. Anorexia/bulimia
  - Strongly oppose
  - Somewhat oppose
  - Neither support nor oppose
  - Somewhat support
  - Strongly support

QX. To what extent do you agree or disagree with the statement that mental health treatments can help people with mental illness lead normal lives?

- Strongly disagree
- Somewhat disagree
- Neither disagree nor agree
- Somewhat agree
- Strongly agree

QX. To what extent do you agree or disagree with the statement that substance disorder treatments can help people with a substance use disorder recover?

- Strongly disagree
- Somewhat disagree
- Neither disagree nor agree
- Somewhat agree
- Strongly agree

QX. What percentage of adults in the United States do you think has a diagnosable mental illness? Your best guess is fine.

- Less than 5%
- 5 to 10%
- 11 to 15%
- 16 to 20%
- 21 to 25%
- 26 to 30%
- More than 30%

QX. What percentage of adults in the United States do you think has a diagnosable substance use disorder? Your best guess is fine.

- Less than 5%
- 5 to 10%
- 11 to 15%
- 16 to 20%
- 21 to 25%
- 26 to 30%
- More than 30%

QX. To what extent do you think that each of the following events, when experienced as a child, increase a person’s risk of developing a mental illness or substance use disorder as an adult?

- QXa. Childhood physical abuse

Little risk increase - 1 2 3 4 5- Major risk increase

- QXb. Childhood sexual abuse

Little risk increase - 1 2 3 4 5- Major risk increase

- QXc. Childhood neglect

Little risk increase - 1 2 3 4 5- Major risk increase

- QXd. Witnessing domestic violence as a child

Little risk increase - 1 2 3 4 5- Major risk increase

QX. Have you heard of the Adverse Childhood Experiences Study (also known as the “ACE Study”)

Yes No Not sure

QX. To what extent do you agree or disagree with the statement that locating a group home or apartment for people with mental illness in a residential neighborhood endangers local residents?

- Strongly disagree
- Somewhat disagree
- Neither disagree nor agree
- Somewhat agree
- Strongly agree

QX. To what extent do you agree or disagree with the statement that people with serious mental illness are, by far, more dangerous than the general public?

- Strongly disagree
- Somewhat disagree
- Neither disagree nor agree
- Somewhat agree
- Strongly agree

QX. To what extent would you be willing or unwilling to have a person with a serious mental illness work closely with you on a job?

- Very unwilling
- Somewhat unwilling
- Neither unwilling nor willing
- Somewhat willing
- Very willing

QX. To what extent would you be willing or unwilling to have a person with a serious mental illness as a neighbor?

- Very unwilling
- Somewhat unwilling
- Neither unwilling nor willing
- Somewhat willing
- Very willing

QX. Leaving yourself aside, have you personally ever known someone who has sought treatment for either of the following?

- A mental health issue
  - Yes
  - No
- A substance abuse issue
  - Yes
  - No

QX. Have you personally ever sought treatment for either of the following?

- A mental health issue
  - Yes
  - No
- A substance abuse issue
  - Yes
  - No

*Next are six questions about how you, as a State Legislator, use mental health/substance abuse research, such as the results of scientific studies (e.g., data on the prevalence of mental health conditions, effective treatments, evidence-based policies).*

Qx. Thinking about the last 12 months, how often, if at all, did you use mental health/substance abuse research in your work as a State Legislator?

Daily Weekly Monthly Quarterly Yearly Never

Qx. If you were going to seek out mental health/substance abuse research to make a policy decision, who would you turn to? Select up to three sources.

- Legislative staff
- University researchers
- Industry sources (e.g., insurance or pharmaceutical companies)
- Advocacy organizations (e.g., National Alliance on Mental Illness)
- Mental health/substance abuse societies (e.g., American Psychological Association)
- Legislator assistance organizations (e.g., National Conference of State Legislatures)
- State mental health/substance abuse agencies
- Other: [open-ended]
- I would not know who to turn to for this research.

Qx. What do you perceive as the biggest barriers, if any, to using mental health/substance abuse research as a state legislator? Select up to three barriers.

- Lack of time to use research
- Lack of access to research
- Research not relevant to my needs
- Research is not available in time to address my needs
- Poor verbal communication of research findings
- Poor visual presentation of research finding
- Lack of training in how to assess research evidence
- Lack of interaction or collaboration with researchers
- Lack of actionable messages/recommendations in written reports and summaries of research
- Lack of clear summary of research findings
- Other [open-ended]
- No barriers

QX. On a scale from 1 to 5, where 1 means not important and 5 means extremely important, please rate the importance of the following characteristics of mental health/substance abuse research.

If you were to receive mental health/substance abuse research, how important would it be, if at all, that the research have each of the following characteristics? Please answer on a scale from 1 to 5, where 1 means not important and 5 means extremely important.

1. It is relevant to my constituents
2. It is delivered by someone I know or respect
3. It tells a story of how an issue affects my constituents
4. It is presented in a brief, concise way
5. It provides data on cost-effectiveness
6. It provides data on budget impact
7. It presents implications that are politically feasible when I receive them

QX. On a scale from 1 to 5, where 1 means not more likely and 5 means much more likely, how much more likely would you be to use a research brief if it presented data about mental health/substance abuse problems among residents in your legislative district instead of your state as a whole?

Not more likely- 1 2 3 4 5- Much more likely

QX. On a scale from 1 to 5, where 1 means not more likely and 5 means much more likely, how much more likely would you be to use a research brief if it presented data about the cost-effectiveness of mental health/substance abuse treatments, instead of just the clinical effectiveness of these treatments.

Not more likely- 1 2 3 4 5- Much more likely

Qx. When a mental health/substance abuse bill is introduced in your state’s legislature, which factors have the most influence on whether or not you support it? Select up to two factors.

- The extent to which the bill is going to impact the state budget
- The extent to which the bill is going to affect my constituents
- The extent to which the bill is based on scientific evidence
- The extent to which the bills is aligned with the values of my political party
- Other(s): _____________________________

*Last are a few questions about you as a state legislator.*

QX. What do you perceive as the most important health issues for legislative action in your state? Select up to three issues.

- Access to healthcare
- Aging
- Cancer
- Diabetes
- Diet/nutrition
- Heart disease
- HIV/AIDS
- Infectious diseases
- Injury prevention
- Medicare/Medicaid
- Mental health
- Obesity
- Physical activity
- Quality of healthcare
- Substance abuse
- The environment
- Tobacco use prevention/cessation
- Other(s) [open-ended]
- Increased access to healthcare
- Violence

QX. When it comes to social issues, do you usually think of yourself as...

- Extremely Liberal
- Liberal
- Slightly Liberal
- Moderate
- Slightly Conservative
- Conservative
- Extremely Conservative

QX. When it comes to fiscal issues, do you usually think of yourself as...

- Extremely Liberal
- Liberal
- Slightly Liberal
- Moderate
- Slightly Conservative
- Conservative
- Extremely Conservative

QX. What is the highest level of education that you have completed?

- Some high school or less
- High school graduate
- Trade, technical, or vocational education beyond high school
- Some college
- College degree
- Postgraduate degree

QX. How many years have you served as a state legislator?

- Less than one
- One to two
- Three to five
- Six to nine
- Ten or more

QX. Are you currently a member of either of the following committees?

- Health committee
  - Yes
  - No
- Insurance committee
  - Yes
  - No

QX. Have you ever introduced a bill that was focused either of the following topics?

- Mental health
  - Yes
  - No
- Substance abuse
  - Yes
  - No

QX.. Please indicate why you took the time to complete this survey. Select up to two reasons.

- I have a personal interest in mental health/substance abuse issues
- I believe that mental health/substance issues affect my constituents
- I want research findings to be more effectively communicated to state legislators
- I value research and believe that it is important to patriciate in studies
- Other: ___________________________

*Thank you very much for your help. If you are interested in the findings from our project, we are glad to provide this information to you when the findings are available. Please provide the e-mail address to which we should send a summary of the survey findings.*

- [open text]
- Do not wish to receive a summary of findings
