# Supplementary material for: Audience segmentation to disseminate behavioral health evidence to legislators: an empirical clustering analysis
Source: Implement Sci. 2018 Sep 19;13:121. doi: 10.1186/s13012-018-0816-8 (PMC6148796; doi:10.1186/s13012-018-0816-8)
Supplement: Supplementary file 2 — Source of behavioral health research across the three audience segments, US state legislators, 2017 (N = 475). (DOCX 48 kb) [file 13012_2018_816_MOESM2_ESM.docx]

**Additional File 2 Source of Behavioral Health Research Across the Three Audience Segments, US State Legislators, 2017 (N = 475)**

|  | **Budget-Oriented Skeptics**  **with Stigma** | **Action-Oriented Supporters** | **Passive Supporters** | **χ^2^**  **p-value** |
| --- | --- | --- | --- | --- |
| **Source** | **%** | **%** | **%** |  |
| Legislative staff | 52.9 | 46.1 | 50.5 | .497 |
| Advocacy organizations (e.g., NAMI) | 35.5 | 69.8 | 57.0 | <.0001 |
| State mental health or substance  abuse agencies | 53.6 | 47.2 | 41.8 | .086 |
| Legislator assistance organizations  (e.g., NCSL) | 37.0 | 39.9 | 30.9 | .301 |
| Mental health/substance abuse societies (e.g., APA) | 28.4 | 36.3 | 30.3 | .332 |
| University researchers | 18.3 | 29.5 | 35.4 | .0009 |
| Industry (e.g., insurance or pharmaceutical companies) | 14.9 | 5.7 | 9.6 | .032 |
| I would not know who to turn to for this research. | 1.4 | 0.0 | 0.6 | .391 |

X^2^ testing differences in the proportion of legislators in each audience segment selecting each source in response to the question: “If you were going to seek out mental health/substance abuse research to make a policy decision, who would you turn to?” Participants were asked to choose up to 3 out of 9 sources. Open-ended response options results not presented

NAMI= National Alliance on Mental Illness, NCSL = National Conference of State Legislatures, APA= American Psychological Association
